# CSF Amyloid-β42 associates with neuropsychiatric and cognitive outcomes via cerebral glucose metabolism

**DOI:** 10.1186/s13041-025-01229-3

**Published:** 2025-07-01

**Authors:** Ali Azargoonjahromi, Hamide Nasiri, for the Alzheimer’s Disease Neuroimaging Initiative#

**Affiliations:** 1 Shiraz University of Medical Sciences, Shiraz, Iran; 2 Student Research Committee, School of Medicine, Zanjan University of Medical Sciences, Zanjan, Iran

**Keywords:** Amyloid-β42, Cerebral glucose metabolism, Neuropsychiatric symptoms, Cognitive performance, Mild cognitive impairment, Alzheimer’s disease

## Abstract

Amyloid-β42 (Aβ42) regulates synaptic plasticity and memory formation at physiological levels in the brain, but in Alzheimer’s disease (AD), it can disrupt brain function and glucose metabolism. This disruption contributes to cognitive decline and neuropsychiatric symptoms, highlighting the need to better understand its complex effects. This study investigated the associations among cerebrospinal fluid (CSF) Aβ42 levels, cerebral glucose metabolism (assessed via FDG-PET), neuropsychiatric symptoms (evaluated using the NPI), and cognitive performance (measured by ADAS-Cog13 and MoCA) in individuals with AD, mild cognitive impairment (MCI), and cognitively normal (CN) participants. After adjusting for age, gender, education, and ApoE ɛ4 status, a significant positive relationship between CSF Aβ42 levels and cerebral glucose metabolism was observed in the MCI and AD groups, but not in the CN group. In the MCI group, higher cerebral glucose metabolism was associated with reductions in both neuropsychiatric and depressive symptoms, suggesting that higher glucose metabolism reflect higher activation state of investigated brain regions. In contrast, in the CN group, elevated CSF Aβ42 levels were directly linked to increased depressive symptoms, indicating that higher CSF Aβ42 may contribute to depression even in the absence of cognitive decline. Further analysis revealed that CSF Aβ42 levels were indirectly associated with reduced neuropsychiatric and depressive symptoms through enhanced cerebral glucose metabolism as mediator solely in the MCI group. Regarding cognitive performance, cerebral glucose metabolism showed a strong relationship with cognition in both the MCI and AD groups. Furthermore, higher CSF Aβ42 levels were positively associated with better cognitive performance in the MCI and AD groups, with cerebral glucose metabolism potentially mediating this relationship, while no effect was seen in the CN group. In short, CSF Aβ42 positively influenced cerebral glucose metabolism, which was linked to reduced neuropsychiatric and depressive symptoms as well as improved cognitive performance in MCI and AD groups.

## Introduction

Amyloid-β42 (Aβ42) is a peptide with a dual role in the brain, where it can be both beneficial and harmful depending on its context. Under normal physiological conditions, Aβ42 is involved in various essential functions, such as synaptic activity, neuronal communication, and possibly even neuroprotection. However, in the context of neurodegenerative diseases like Alzheimer’s disease (AD), Aβ42 becomes problematic. When it accumulates and forms amyloid plaques in the brain, it disrupts normal brain function, leading to neuroinflammation, oxidative stress, and neuronal damage, which contribute to cognitive decline and dementia [1, 2].

One way that accumulation of Aβ42 affects dementia is by disrupting cerebral glucose metabolism. This disruption impairs the brain’s ability to properly utilize glucose as a primary energy source, leading to energy deficits that hinder normal cognitive functions. As a result, the brain struggles to maintain synaptic plasticity and neuronal survival, accelerating neurodegeneration [3–6]. However, the role of soluble cerebrospinal fluid (CSF) Aβ42 in cognitive improvement is still under investigation, and its effects on cerebral glucose metabolism need further investigations to clarify the underlying mechanisms and potential implications for neurodegenerative diseases.

In addition to symptoms such as memory loss, a common feature of neurodegenerative diseases is the presence of neuropsychiatric symptoms, including hallucinations, delusions, depression, anxiety, and apathy. These symptoms often co-occur with cognitive decline and can greatly impact the quality of life, making daily functioning more challenging for affected individuals and their caregivers [7–10]. Recent studies have shown that CSF Aβ42 levels can influence mood and neuropsychiatric symptoms in patients with AD [11–13]. Notably, some studies have found that cerebral glucose metabolism can affect neuropsychiatric symptoms [14, 15], but the mechanisms by which impaired glucose metabolism leads to these changes, or whether Aβ42 in the CSF can directly affect these processes, remain unclear. Further research is needed to better understand the complex relationship between glucose metabolism, amyloid pathology, and the onset or progression of neuropsychiatric symptoms in neurodegenerative diseases.

Despite the aforementioned findings, a substantial gap persists in understanding the specific influence of CSF Aβ42 on neuropsychiatric symptoms and cognitive functions. The complex interplay between CSF Aβ42, cerebral glucose metabolism, neuropsychiatric manifestations, and cognitive processes warrants further investigation to elucidate its role in symptomatology and disease progression. Thus, this study aimed to investigate the potential links between CSF Aβ42 levels, cerebral glucose metabolism measured by FDG-PET, neuropsychiatric symptoms, and cognitive performance in patients with AD, mild cognitive impairment (MCI), and cognitively normal (CN) individuals, with a focus on elucidating the underlying mechanisms that may influence mood, cognition, and disease progression.

## Methods and materials

This study utilized data from the Alzheimer’s Disease Neuroimaging Initiative (ADNI) database (www.adni.loni.usc.edu). Founded in 2003 and led by Dr. Michael W. Weiner, ADNI aims to improve our understanding of MCI and early AD by exploring how MRI, PET imaging, biomarkers, and neuropsychological tests can track disease progression.

### Cognitive assessment

Cognitive status in participants was evaluated using two key tools: The Alzheimer’s Disease Assessment Scale-Cognitive Subscale (ADAS-Cog) [16, 17] and Montreal Cognitive Assessment (MoCA) [18]. The ADAS-Cog, widely used for monitoring cognitive decline in conditions such as MCI and AD, measures cognitive impairment through tasks like word recall, object naming, and geometric figure copying. Scores range from 0 to 70, with higher scores indicating greater impairment. The ADAS-Cog 13 is a more detailed version, allowing for a comprehensive assessment of cognitive function [17].

The MoCA is a brief screening tool used to evaluate cognitive function and detect MCI and early AD. It assesses various cognitive domains, including memory, attention, language, executive function, and visuospatial abilities, through tasks like short-term memory recall, clock drawing, and attention tests. Scored from 0 to 30, a score below 26 suggests potential cognitive impairment. MoCA is valuable for identifying subtle cognitive changes in the early stages of neurodegenerative diseases [18, 19].

Participants with major neurological or psychiatric disorders, substance abuse, or conditions affecting cognition, were excluded from participating. This classification follows the ADNI protocol, allowing the ability to track cognitive decline and neurodegeneration.

### CSF Amyloid-β (Aβ) measurement

CSF Aβ42 was quantified using a validated and highly specific two-dimensional ultra-performance liquid chromatography tandem mass spectrometry (2D-UPLC-MS/MS) method. This method began with the treatment of CSF samples with highly concentrated guanidine HCl to denature proteins and release amyloid-beta peptides. These peptides were then isolated from other endogenous compounds using mixed-mode ion-exchange microelution columns. Calibration standards and quality controls were prepared using a surrogate matrix composed of bovine serum albumin in an artificial CSF electrolyte solution to ensure reproducibility and accuracy.

The measurements were performed on a Waters XEVO TQ-S mass spectrometer, which monitored specific precursor-to-fragment ion transitions for Aβ1–42 (m/z 1129.6 → 1079.0) and other Aβ peptides. A reference standard for Aβ1–42, provided by the EC-JRC-Institute for Reference Materials and Measurements (IRMM), ensured precision, with concentrations validated against assigned values. Each CSF sample was analyzed in duplicate using 0.1 mL aliquots, and the reported value was the average of these measurements to minimize variability. A cut-off value of 192 pg/mL for CSF Aβ42 was used to define pathological amyloid levels, consistent with ADNI-established thresholds measured using the INNO-BIA AlzBio3 immunoassay. Although our study used a mass spectrometry-based method (2D-UPLC-MS/MS) for quantification, this cut-off provides a commonly accepted benchmark for amyloid pathology classification.

### Cerebral glucose metabolism assessment by Fluorodeoxyglucose (FDG) positron emission tomography (PET) scan

In this study, FDG-PET was measured using a set of predefined regions of interest (MetaROIs) that were derived from a comprehensive meta-analysis of FDG-PET studies involving AD and MCI. The PET data was downloaded from the Laboratory of Neuro Imaging (LONI) in its most processed form (Co-reg, Avg, Std Img, and Vox Siz, Uniform Resolution). These images were spatially normalized to the MNI PET template using statistical parametric mapping (SPM), which allows for consistent anatomical alignment across subjects.

The FDG uptake was quantified by extracting the mean intensity values from the MetaROIs, which represent brain regions most commonly associated with hypometabolism in AD and MCI. The MetaROIs were generated by reviewing cross-sectional and longitudinal studies that identified significant metabolic changes in these conditions. The five key MetaROIs included the left and right angular gyri, bilateral posterior cingulate gyrus, and the left and right inferior temporal gyrus, regions known to show abnormal glucose metabolism in AD and MCI. These regions were binarized and combined into a single composite MetaROI for analysis.

Additionally, to account for individual variability in brain metabolism, the mean intensity of the MetaROIs was intensity-normalized by dividing by the mean FDG uptake of the top 50% of voxels within a hand-drawn reference region in the pons/cerebellar vermis, which served as a reference for normal brain metabolism. The final FDG-PET values were intensity-normalized and then analyzed to identify hypometabolic regions linked to cognitive decline. The method allowed for a detailed and reliable quantification of FDG uptake, facilitating comparisons across subjects with varying levels of cognitive function. Since FDG-PET reflects cerebral glucose metabolism, the term FDG-PET was used to denote cerebral glucose metabolism in this study.

### Neuropsychiatric inventory (NPI)

The Neuropsychiatric Inventory (NPI) is a comprehensive tool designed to assess behavioral and psychological symptoms in individuals with dementia and other neuropsychiatric disorders. It evaluates 12 symptom domains, including delusions, hallucinations, agitation, depression, anxiety, euphoria, apathy, disinhibition, irritability, aberrant motor behavior, sleep disturbances, and appetite changes. The assessment is conducted through a structured interview with a caregiver or informant familiar with the patient, measuring both the severity (on a 1–3 scale) and frequency (on a 1–4 scale) of each symptom. Composite scores for each symptom are calculated by multiplying severity by frequency, and the total score reflects the overall neuropsychiatric burden. Widely used in clinical practice and research, the NPI is instrumental in monitoring symptom progression and evaluating treatment outcomes in conditions such as AD and other dementias [20, 21].

The depression subscore from the NPI can assess depressive symptoms in participants. It is calculated by multiplying severity (1–3) and frequency (1–4) ratings. In this study, the NPI total score was used to evaluate the overall neuropsychiatric symptom burden, and the NPI depression subscore (NPID) was used to specifically assess depressive symptoms.

### Statistical analysis

Statistical analyses were performed using SPSS version 27. Outliers based on standard deviations were removed to ensure the robustness of the analysis. Descriptive statistics (mean, standard deviation, frequencies, and percentages) were used to summarize baseline demographic characteristics of the study participants. ANOVA was applied to compare continuous variables (age, ADAS-Cog 13, MoCA, and CSF Aβ42 levels) across the CN, MCI, and AD groups, while chi-square tests were used for categorical variables (gender, ApoE ɛ4 carrier status) to assess group differences. Post-hoc Tukey’s HSD tests were conducted following significant ANOVA results to further explore pairwise group differences. Pearson’s correlation analysis was conducted to assess the relationships between NPI and NPID symptoms and cognitive functions (ADAS-Cog 13 and MoCA), adjusting for confounding variables such as age, gender, education, and ApoE ɛ4 status. Mediation analysis was performed using a bootstrapping method (5000 resamples), and all models controlled for potential confounders including age, gender, education, and ApoE ɛ4 status, and the significance level was set at *p* < 0.05 for all analyses.

## Results and discussion

In this study, 642 participants were recruited and divided into three groups based on their cognitive status: 238 were classified as CN, 289 as having MCI, and 115 as having AD. The baseline characteristics reveal significant differences across the CN, MCI, and AD groups.

### Characteristics of participants

AD participants were older than CN and MCI participants (*P* = 0.016) and had fewer years of education (*P* = 0.003). Gender distribution differed significantly (*P* = 0.031), with a higher proportion of males in the MCI and AD groups. ApoE ɛ4 carrier status was more frequent in the MCI (21.8%) and AD (16.5%) groups compared to CN (9.7%) (*P* < 0.001). Cognitive performance, as measured by ADAS-Cog 13 and MoCA scores, showed progressive decline from CN to MCI to AD (*P* < 0.001 for both). Similarly, CSF Aβ42 levels were significantly lower in the AD group compared to CN and MCI groups (*P* < 0.001). (Table 1)

**Table 1:** Baseline demographic characteristics of participants

	CN (*N* = 238)	MCI (*N* = 289)	AD (*N* = 115)	*P* value
**Age**, **year**	69.13 (6.27)	68.07 (7.34)	70.30 (8.63)	**0.016** ^a^
**Gender**				**0.031** ^b^
Male	110 (46.2%)	161 (55.7%)	68 (59.1%)	
Female	128 (53.8%)	128 (44.3%)	47 (40.9%)	
**Education**, **year**	16.68 (2.51)	16.34 (2.61)	15.65 (2.71)	**0.003** ^a^
**ApoE** ɛ**4 carrier**				**< 0.001** ^b^
Negative	215 (90.3%)	226 (78.2%)	96 (83.5%)	
Positive	23 (9.7%)	63 (21.8%)	19 (16.5%)	
**ADAS-Cog 13**	8.92 (4.38)	15.75 (7.23)	29.86 (9.70)	**< 0.001** ^a^
**MoCA**	7.94 (0.86)	7.59 (1.03)	6.30 (1.43)	**< 0.001** ^**a**^
**CSF Aβ42**	1462.58 (648.91)	1110.78 (534.43)	823.29 (460.12)	**< 0.001** ^a^

**Abbreviations**: **CN**, Cognitively Normal; **MCI**, Mild Cognitive Impairment; **AD**, Alzheimer’s Disease; **ADAS-Cog 13**, Alzheimer’s Disease Assessment Scale – Cognitive Subscale (13 items); **CSF Aβ42**: CSF Amyloid-β42; **MoCA**: Montreal Cognitive Assessment; **ApoE** ɛ**4 carrier**, Presence of Apolipoprotein E epsilon 4 allele; Continuous variables are reported as Mean (Standard Deviation); Categorical variables are reported as Number (Percentage); ^**a**^ ANOVA test; ^**b**^ Chi-Square test. Bold values indicate statistical significance

The significant reduction in CSF Aβ42 levels observed in our study aligns with these established findings, reinforcing the peptide’s role as a key biomarker in AD diagnosis and progression. This decrease can be primarily attributed to the aggregation of Aβ42 into amyloid plaques within the brain, leading to reduced soluble Aβ42 in the CSF. Studies have consistently demonstrated that AD patients exhibit lower CSF Aβ42 levels compared to CN individuals, with MCI patients displaying intermediate levels [22–26]. Furthermore, the CSF Aβ42/40 ratio has been identified as a more accurate biomarker for AD diagnosis. Research indicated that this ratio is significantly decreased in AD patients compared to non-AD individuals, enhancing diagnostic specificity [23, 26–28].

Aβ42 is a hydrophobic peptide produced through the cleavage of APP by β- and γ-secretases. Highly prone to aggregation, Aβ42 forms insoluble amyloid plaques in the extracellular spaces of the brain, a hallmark of AD. As the disease progresses, Aβ42 aggregates into plaques within the brain parenchyma, sequestering it from the soluble pool. This aggregation reduces the amount of Aβ42 that can diffuse into the interstitial fluid and subsequently into the CSF, resulting in a progressive decline in soluble Aβ42 concentration, with the extent of this reduction correlating with the accumulation of amyloid plaques as AD advances [1, 29, 30].

Notably, altered clearance mechanisms contribute to the reduction of CSF Aβ42 in AD. Normally, Aβ42 is cleared via the glymphatic system, enzymatic degradation, and receptor-mediated transport, but these pathways are impaired in AD, leading to increased plaque deposition in the brain [1, 31, 32]. Genetic factors, such as mutations in amyloid precursor protein (APP) or presenilin genes, further exacerbate Aβ42 aggregation into plaques. Thus, this diminution in CSF Aβ42 reflects the redistribution of the peptide into brain deposits, serving as an indirect marker of amyloid plaque burden and aiding in AD diagnosis and progression monitoring [32, 33].

### Associations between CSF Amyloid-β42, cerebral glucose metabolism (FDG-PET), and depression symptoms (NPID)

The study revealed a significant positive association between CSF Aβ42 levels and cerebral glucose metabolism (FDG-PET) in individuals with MCI (β = 0.177, *p* < 0.001) and AD (β = 0.212, *p* = 0.004), but not in CN individuals (β = 0.066, *p* = 0.256). (Table 2)

**Table 2:** Association of CSF Amyloid-β42 with glucose metabolism (Measured by FDG-PET) and depression symptoms (Measured by NPID)

	CN (*N* = 238)		MCI (*N* = 289)		AD (*N* = 115)	
	β (S.E.)	*P* value	β (S.E.)	*P* value	β (S.E.)	*P* value
FDG-PET ∼ CSF Aβ42	0.066 (0.058)	0.256	0.177 (0.051)	**< 0.001**	0.212 (0.073)	**0.004**
NPID ∼ FDG-PET	0.089 (0.074)	0.227	-0.156 (0.072)	**0.031**	-0.098 (0.099)	0.325
Total	0.139 (0.066)	**0.035**	-0.068 (0.063)	0.282	-0.111 (0.095)	0.246
Direct	0.133 (0.066)	**0.044**	-0.040 (0.064)	0.528	-0.098 (0.099)	0.325
Indirect	0.006 (0.009)	[-0.0069, 0.0304]	-0.027 (0.014)	**[-0.0625**, -0.0047]^*^	-0.013 (0.025)	[-0.0647, 0.0395]

**Abbreviations**: **CN**, Cognitively Normal; **MCI**, Mild Cognitive Impairment; **AD**, Alzheimer’s Disease; **NPID**: Depression Symptoms Score; **FDG-PET**: Measured Cerebral Glucose Metabolism; **CSF Aβ42**: CSF Amyloid-β42. It was adjusted for age, gender, education, and ApoE ɛ4 status. * [BootLLCI, BootULCI]: For the Indirect Effect, the *p*-value is based on the 95% bootstrap confidence interval that does not include zero. Bold values indicate statistical significance

These findings are consistent with prior research linking CSF Aβ42 levels to cerebral glucose metabolism [34–39]. In MCI and AD, amyloid plaques disrupt synaptic function and neuronal communication, triggering compensatory metabolic changes, such as an increase in cerebral glucose metabolism, as the brain tries to maintain cognitive function despite neuronal dysfunction. This compensatory mechanism is most pronounced in the earlier stages of neurodegeneration—MCI—and the more advanced stages—AD, where cerebral glucose metabolism increases to meet the higher energy demands of dysfunctional neurons. The lack of such a relationship in the CN group supports the idea that amyloid deposition alone, without cognitive decline, does not substantially affect brain metabolism [40–42]. These findings highlighted how amyloid accumulation leads to metabolic alterations, particularly in the context of neurodegenerative diseases, and emphasized the role of compensatory mechanisms in the early stages of neurodegeneration.

In terms of depression symptoms, the study found a significant negative association between FDG-PET and NPID in the MCI group (β = -0.156, *p* = 0.031), indicating that higher cerebral glucose metabolism might alleviate depressive symptoms in individuals with MCI. This finding is consistent with prior research highlighting the role of brain metabolism in mood regulation. Increased cerebral glucose metabolism in regions like the prefrontal cortex, a key area involved in mood control, may act as a compensatory mechanism to mitigate depressive symptoms, particularly during the MCI stage [43–45].

However, the study found no significant relationship between FDG-PET and depression in either the CN (β = 0.089, *p* = 0.227) or AD (β = -0.098, *p* = 0.325) groups. In CN individuals, depressive symptoms may be influenced more by psychosocial or peripheral factors rather than direct brain metabolic changes [46, 47]. In the AD group, severe neurodegeneration and widespread cerebral glucose hypometabolism might overshadow or complicate the metabolic patterns specifically associated with mood regulation [48–50]. This highlighted the complexity of depression’s interaction with brain metabolism, suggesting it may vary depending on cognitive status and disease progression.

Regarding the total and direct effects of CSF Aβ42 on depression symptoms, the study found a significant positive association between CSF Aβ42 levels and depression symptoms in the CN group (β = 0.139, *p* = 0.035), primarily driven by a direct effect (β = 0.133, *p* = 0.044). This is a notable finding, suggesting that elevated CSF Aβ42 levels may influence depression symptoms even in the absence of cognitive decline.

Nonetheless, one study explored the longitudinal effects of biomarkers and found that higher CSF tau/Aβ42 levels were associated with greater increases in depressive symptoms over time in cognitively normal older adults. These results do not contradict the idea that amyloid-β may have varying effects at different stages of neurodegeneration, as individuals with higher tau/Aβ42 ratios experienced more significant mood disturbances, including depression, during the follow-up period [51]. In contrast, another study found that at the screening stage, plasma Aβ42/Aβ40 levels did not correlate with depression severity (measured by the Hamilton Depression Scale) or cognitive performance (measured by the Mini-Mental State Examination). However, after adjusting for factors like age, education, cognitive performance, and antidepressant use, Aβ42/Aβ40 levels were linked to depression severity at a 3-year follow-up [52].

Additionally, a separate study reported no significant correlation between Aβ burden and depressive symptom severity in patients with late-onset depression [53]. Notably, a recent meta-analysis also found no evidence of an association between depression or depressive symptoms and Aβ levels, whether measured in CSF, PET, or other methods [54]. This discrepancy may be due to differences in study design, sample populations, or methodologies. The current study’s findings highlight the complexity of the relationship between CSF Aβ42 and depression, suggesting that this association may be more pronounced in the early stages of cognitive health and may vary across different populations.

Importantly, the study observed a significant indirect effect of CSF Aβ42 on depression symptoms through FDG-PET solely in the MCI group (β = -0.027, CI = [-0.0625, -0.0047]), indicating that in MCI individuals, CSF Aβ42 levels showed a negative association with depressive symptoms, and this relationship may be mediated by cerebral glucose metabolism. This result supported the hypothesis that changes in brain metabolism might mediate the relationship between Aβ and mood disturbances in the early stages of cognitive decline [55].

Several factors may explain the finding that CSF Aβ42 impacts depressive symptoms through brain metabolism in individuals with MCI. In the early stages of cognitive decline, elevated Aβ42 levels could reflect a period before amyloid deposition, where enhanced brain glucose metabolism indicates better function, possibly alleviating depressive symptoms. FDG-PET, which tracks brain activity, suggests that increased metabolism may help compensate for early cognitive decline, providing a buffer against depression. However, as neurodegeneration advances, this compensatory mechanism diminishes, resulting in more pronounced cognitive and mood disturbances.

### Associations between CSF Amyloid-β42, cerebral glucose metabolism (FDG-PET), and neuropsychiatric symptoms (NPI)

The study investigated the relationship between CSF Aβ42, glucose metabolism measured by FDG-PET, and neuropsychiatric symptoms (NPI), while adjusting for key confounding factors including age, gender, education, and ApoE ɛ4 status. (Table 3)

**Table 3:** Association of CSF Amyloid-β42 with glucose metabolism (Measured by FDG-PET) and neuropsychiatric symptoms (Measured by NPI)

	CN (*N* = 238)		MCI (*N* = 289)		AD (*N* = 115)	
	β (S.E.)	*P* value	β (S.E.)	*P* value	β (S.E.)	*P* value
FDG-PET ∼ CSF Aβ42	0.066 (0.058)	0.256	0.177 (0.052)	**< 0.001**	0.212 (0.073)	**0.004**
NPI ∼ FDG-PET	0.109 (0.074)	0.141	-0.190 (0.071)	**0.007**	-0.041 (0.121)	0.736
Total	0.024 (0.066)	0.717	-0.097 (0.062)	0.118	0.079 (0.092)	0.391
Direct	0.016 (0.066)	0.801	-0.064 (0.063)	0.309	0.088 (0.096)	0.361
Indirect	0.007 (0.009)	[-0.0067, 0.0297]	-0.034 (0.014)	**[-0.0642**, -0.0091]^*^	-0.008 (0.021)	[-0.0534, 0.0318]

**Abbreviations**: **CN**, Cognitively Normal; **MCI**, Mild Cognitive Impairment; **AD**, Alzheimer’s Disease; **NPI**: Neuropsychiatric Symptoms Score; **FDG-PET**: Measured Cerebral Glucose Metabolism; **CSF Aβ42**: CSF Amyloid-β42. It was adjusted for age, gender, education, and ApoE ɛ4 status. * [BootLLCI, BootULCI]: For the Indirect Effect, the *p*-value is based on the 95% bootstrap confidence interval that does not include zero. Bold values indicate statistical significance

The findings revealed a significant positive association between CSF Aβ42 and FDG-PET, particularly in individuals with MCI and AD. In both MCI and AD, CSF Aβ42 was positively associated with glucose metabolism, with β = 0.177 (*p* < 0.001) in MCI and β = 0.212 (*p* = 0.004) in AD. In contrast, no significant relationship between Aβ42 and FDG-PET was observed in CN individuals (β = 0.066, *p* = 0.256).

Regarding neuropsychiatric symptoms, a significant negative association was found between FDG-PET and NPI in the MCI group (β = -0.190, *p* = 0.007), suggesting that better cerebral glucose metabolism was associated with fewer neuropsychiatric symptoms. However, no significant relationship was observed in the AD (β = -0.041, *p* = 0.736) or CN (β = 0.109, *p* = 0.141) groups. This finding aligns with previous studies linking neuropsychiatric symptoms, such as hallucinations, delusions, apathy, or anxiety, to cerebral glucose metabolism [56, 57]. The current study extends this by showing that in the MCI stage, better glucose metabolism is associated with fewer neuropsychiatric symptoms. However, in contrast to former studies [58–61], this relationship was not observed in the AD.

Importantly, a significant negative indirect effect was identified in the MCI group (β = -0.034, *p* < 0.05), indicating that CSF Aβ42 may reduce neuropsychiatric symptoms through its role in enhancing cerebral glucose metabolism in individuals with MCI; this mediation effect was not evident in the CN or AD groups. This suggests that in MCI, the brain retains compensatory mechanisms that help preserve neuronal function and emotional stability, allowing cerebral glucose metabolism to play a role in alleviating psychiatric symptoms.

### Associations between neuropsychiatric (NPI) and depression (NPID) symptoms and cognitive functions (ADAS-Cog 13 and MoCA)

The analysis revealed that neuropsychiatric (NPI) and depression (NPID) symptoms showed different associations with cognitive functions (ADAS-Cog 13 and MoCA) across CN, MCI, and AD groups, with results consistent after adjusting for age, gender, education, and ApoE ɛ4 status. (Table 4)

**Table 4:** Association of neuropsychiatric (NPI) and depression (NPID) symptoms and cognitive functions (ADAS-Cog 13 and MoCA)

	CN (*N* = 238)		MCI (*N* = 289)		AD (*N* = 115)	
	β (S.E.)	*P* value	β (S.E.)	*P* value	β (S.E.)	*P* value
			**ADAS-Cog 13**			
NPI	-0.011 (0.05)	0.863	0.156 (0.07)	**0.008**	0.033 (0.08)	0.728
NPID	-0.093 (0.05)	0.158	0.171 (0.07)	**0.004**	-0.129 (0.09)	0.178
			**MoCA**			
NPI	-0.020 (0.05)	0.757	0.016 (0.06)	0.783	0.024 (0.10)	0.804
NPID	-0.078 (0.04)	0.233	-0.013 (0.06)	0.830	0.045 (0.08)	0.638

**Abbreviations**: **CN**, Cognitively Normal; **MCI**, Mild Cognitive Impairment; **AD**, Alzheimer’s Disease; **NPI**: Neuropsychiatric Symptoms Score; **NPID**: Depression Symptoms Score; **ADAS-Cog 13**, Alzheimer’s Disease Assessment Scale – Cognitive Subscale (13 items); **MoCA**: Montreal Cognitive Assessment. It was adjusted for age, gender, education, and ApoE ɛ4 status. Bold values indicate statistical significance

In the MCI group, both NPI and NPID were significantly positively associated with cognitive performance on the ADAS-Cog 13 (β = 0.156, *p* = 0.008; β = 0.171, *p* = 0.004, respectively), suggesting that neuropsychiatric symptoms, including depression, are linked to cognitive impairment and may exacerbate cognitive impairment in this stage. However, in the CN and AD groups, neither NPI nor NPID showed significant associations with ADAS-Cog 13 or MoCA scores, suggesting that these symptoms may have a more pronounced effect on cognitive function during the early stages of cognitive decline like MCI, rather than in the later stages of dementia or in those who are CN.

These findings suggest that neuropsychiatric symptoms in the MCI stage may serve as early indicators of cognitive impairment and could potentially exacerbate cognitive impairment. The presence of these symptoms likely reflects early disturbances in brain function that may worsen cognitive impairment before more substantial neurodegeneration occurs [62–64].

### Associations between CSF Amyloid-β42, cerebral glucose metabolism (FDG-PET), and cognitive functions (ADAS-Cog 13 and MoCA)

The analysis revealed significant associations between CSF Aβ42, cerebral glucose metabolism (measured by FDG-PET), and cognitive performance, particularly in the MCI and AD groups. (Table 5)

**Table 5:** Association of CSF Amyloid-β42 with glucose metabolism (Measured by FDG-PET) and cognitive functions (ADAS-Cog 13 and MoCA)

	CN (*N* = 238)		MCI (*N* = 289)		AD (*N* = 115)	
	β (S.E.)	*P* value	β (S.E.)	*P* value	β (S.E.)	*P* value
			**ADAS-Cog 13**			
FDG-PET ∼ CSF Aβ42	0.066 (0.058)	0.256	0.177 (0.051)	**< 0.001**	0.177 (0.060)	**< 0.001**
ADAS-Cog 13 ∼ FDG-PET	0.001 (0.065)	0.980	-0.182 (0.055)	**< 0.001**	-0.182 (0.060)	**< 0.001**
Total	-0.110 (0.058)	0.059	-0.247 (0.057)	**< 0.001**	-0.247 (0.060)	**< 0.001**
Direct	-0.110 (0.058)	0.060	-0.182 (0.055)	**< 0.001**	-0.182 (0.060)	**< 0.001**
Indirect	0.000 (0.006)	[-0.0142, 0.0133]	-0.064 (0.212)	**[-0.1127**, -0.0291]^*^	-0.064 (0.042)	**[-0.1127**, -0.0291]^*^
			**MoCA**			
FDG-PET ∼ CSF Aβ42	0.066 (0.058)	0.256	0.177 (0.051)	**< 0.001**	0.230 (0.073)	**0.004**
MoCA ∼ FDG-PET	0.015 (0.064)	0.814	0.167 (0.068)	**0.015**	0.501 (0.109)	**< 0.001**
Total	0.008 (0.064)	0.891	0.086 (0.060)	0.150	0.112 (0.092)	0.226
Direct	0.015 (0.064)	0.814	0.057 (0.060)	0.347	-0.003 (0.087)	0.970
Indirect	-0.006 (0.009)	[-0.0314, 0.0056]	0.029 (0.017)	**[0.0021**, 0.0691]^*^	0.115 (0.040)	**[0.0428**, 0.1985]^*^

**Abbreviations**: **CN**, Cognitively Normal; **MCI**, Mild Cognitive Impairment; **AD**, Alzheimer’s Disease; **FDG-PET**: Measured Cerebral Glucose Metabolism; **CSF Aβ42**: CSF Amyloid-β42; **ADAS-Cog 13**, Alzheimer’s Disease Assessment Scale – Cognitive Subscale (13 items); **MoCA**: Montreal Cognitive Assessment. It was adjusted for age, gender, education, and ApoE ɛ4 status. * [BootLLCI, BootULCI]: For the Indirect Effect, the *p*-value is based on the 95% bootstrap confidence interval that does not include zero. Bold values indicate statistical significance

In contrast to the CN group, where the relationship between CSF Aβ42 and FDG-PET was not significant (β = 0.066, *p* = 0.256), the association was stronger in the MCI and AD groups (β = 0.177, *p* < 0.001 and β = 0.230, *p* = 0.004, respectively). These findings suggested that CSF Aβ42 may be a less reliable marker for glucose metabolism in cognitively normal individuals but serves as a more accurate indicator in those with cognitive impairments, particularly in MCI and AD.

Regarding cognitive performance, FDG-PET showed a negative association with ADAS-Cog 13 scores in both MCI (β = -0.182, *p* < 0.001) and AD (β = -0.182, *p* < 0.001), but not in the CN group (β = 0.001, *p* = 0.980). For MoCA scores, FDG-PET was positively associated with cognitive performance in both MCI (β = 0.167, *p* = 0.015) and AD (β = 0.501, *p* < 0.001), but not in CN (β = 0.015, *p* = 0.814).

The negative association between FDG-PET and cognitive performance (ADAS-Cog 13) in the MCI and AD groups, but not in the CN group, is consistent with a substantial body of research that links reduced brain glucose metabolism with worse cognitive outcomes. FDG-PET, which measures regional brain glucose metabolism, has been widely used to assess neuronal activity, but recent studies suggest that glial cells are also involved in the strength of the FDG-PET signal [65]. Studies have consistently shown that in AD and other neurodegenerative conditions, there is a decline in glucose metabolism in areas crucial for memory and cognition, such as the temporoparietal cortex and posterior cingulate [66–71]. As cognitive decline progresses in MCI and AD, these metabolic changes become more pronounced and are associated with impairments in cognitive performance.

Furthermore, the indirect effect of CSF Aβ42 on cognitive function, as measured by ADAS-Cog 13, through FDG-PET was significantly negative in both MCI (β = -0.064, CI = [-0.1127, -0.0291]) and AD (β = -0.064, CI = [-0.1127, -0.0291]), while no significant effect was observed in the CN group. Similarly, the indirect effect of CSF Aβ42 on cognitive function, as measured by MoCA, was positively significant in MCI (β = 0.029, CI = [0.0021, 0.0691]) and AD (β = 0.115, CI = [0.0428, 0.1985]), further emphasizing its mediating role.

These results suggest that higher CSF Aβ42 levels may be linked to better cognitive performance through their positive correlation with cerebral glucose metabolism in MCI and AD, highlighting its potential as a therapeutic target for preserving cognitive function through metabolic interventions. Future research should focus on refining these biomarkers for early detection and intervention in MCI and AD, and further explore their potential in therapeutic settings to slow or prevent cognitive decline.

To the best of available evidence, this was the first study to report a potential indirect association between CSF Aβ42 and cognitive performance mediated by cerebral glucose metabolism, although the correlational nature of the data precludes causal inference. These findings provide preliminary evidence for a possible metabolic pathway linking CSF Aβ42 to cognitive function in MCI and AD. Further longitudinal and mechanistic research is warranted to validate this relationship and elucidate underlying biological mechanisms.

### Limitations

The study had several limitations, including its cross-sectional design, which restricted the ability to make causal inferences or track changes over time. Additionally, the sample size was imbalanced, with a higher proportion of MCI participants compared to those with AD, which may have affected the robustness of the findings. Genetic confounding factors were not fully addressed, potentially influencing the results. The study also relied on standard cognitive assessments, which may not have captured the full spectrum of cognitive decline, and lacked detailed neuroimaging data or consideration of treatment effects, further limiting its comprehensive assessment of neurodegeneration. Moreover, although the Aβ42/Aβ40 ratio is a more reliable biomarker, this study used Aβ42 alone due to limitations in the ADNI database, where ratio data could not be merged with other key variables at the same time point. Future studies should aim to integrate Aβ42/Aβ40 ratio data with neuropsychiatric, cognitive, and metabolic measures to enable a more comprehensive analysis.

## Conclusion

This study aimed to explore the relationship between CSF Aβ42 levels, cerebral glucose metabolism, and neuropsychiatric symptoms in patients with AD, MCI, and CN individuals. The main findings revealed a significant positive association between CSF Aβ42 levels and cerebral glucose metabolism, measured by FDG-PET, in the MCI and AD groups, but not in the CN group. Additionally, the study found a significant negative relationship between cerebral glucose metabolism and neuropsychiatric symptoms, specifically depression symptoms, in the MCI group, suggesting that increased brain metabolism may help alleviate depressive symptoms. Importantly, in individuals with MCI, higher CSF Aβ42 levels were associated with better cognitive performance and fewer neuropsychiatric symptoms, potentially mediated by increased cerebral glucose metabolism. In both MCI and AD groups, CSF Aβ42 levels were positively associated with cognitive performance through their relationship with cerebral glucose metabolism. These findings underscored the importance of cerebral glucose metabolism as a mediator of CSF Aβ42, suggesting that metabolic changes play a key role in modulating cognitive and mood outcomes in neurodegenerative diseases like AD and MCI.

## Data Availability

The data used in this research was obtained from Alzheimer’s Disease Neuroimaging Initiative (ADNI) and is available with permission to all researchers.

## Abbreviations

Aβ42: Amyloid-β42
AD: Alzheimer’s disease
CSF: Cerebrospinal Fluid
MCI: Mild Cognitive Impairment
CN: Cognitively Normal
APP: Amyloid Precursor Protein
FDG-PET: Measured Cerebral Glucose Metabolism
NPI: Neuropsychiatric Inventory (Symptoms)
NPID: Neuropsychiatric Inventory (Subscore Depression Symptoms)
